# Actigraphy as a diagnostic aid for REM sleep behavior disorder in Parkinson’s disease

**DOI:** 10.1186/1471-2377-14-76

**Published:** 2014-04-06

**Authors:** Maartje Louter, Johan BAM Arends, Bastiaan R Bloem, Sebastiaan Overeem

**Affiliations:** 1 Department of Neurology, Donders Institute for Brain, Cognition and Behaviour, Radboud University Nijmegen Medical Centre, PO Box 9101, 6500 Nijmegen, HB, The Netherlands; 2 Sleep Medicine Centre Kempenhaeghe, Heeze, The Netherlands; 3 Epilepsy Centre Kempenhaeghe, Heeze, The Netherlands; 4 Department of Electrical Engineering, Eindhoven University of Technology, Eindhoven, The Netherlands

**Keywords:** Parkinson’s disease, Actigraphy, REM sleep behavior disorder, Polysomnography

## Abstract

**Background:**

Rapid eye movement (REM) sleep behavior disorder (RBD) is a common parasomnia in Parkinson’s disease (PD) patients. The current International Classification of Sleep Disorders (ICSD-II) requires a clinical interview combined with video polysomnography (video-PSG) to diagnose. The latter is time consuming and expensive and not always feasible in clinical practice. Here we studied the use of actigraphy as a diagnostic tool for RBD in PD patients.

**Methods:**

We studied 45 consecutive PD patients (66.7% men) with and without complaints of RBD. All patients underwent one night of video-PSG and eight consecutive nights of actigraphy. Based on previous studies, the main outcome measure was the total number of bouts classified as “wake”, compared between patients with (PD + RBD) and without RBD (PD- RBD).

**Results:**

23 (51.1%) patients had RBD according to the ICSD-II criteria. The total number of wake bouts was significantly higher in RBD patients (PD + RBD 73.2 ± 40.2 vs. PD-RBD 48.4 ± 23.3, p = .016). A cut off of 95 wake bouts per night resulted in a specificity of 95.5%, a sensitivity of 20.1% and a positive predictive value of 85.7%. Seven patients were suspected of RBD based on the interview alone, but not confirmed on PSG; six of whom scored below 95 wake bouts per night on actigraphy.

**Conclusion:**

PD patients with RBD showed a significantly higher number of bouts scored as “wake” using actigraphy, compared to patients without RBD. In clinical practice, actigraphy has a high specificity, but low sensitivity in the diagnosis of RBD. The combination of actigraphy and previously reported RBD questionnaires may be a promising method to diagnose RBD in patients with PD.

## Background

Rapid eye movement (REM) sleep behavior disorder (RBD) is a parasomnia that occurs frequently in patients with Parkinson’s disease (PD), with an estimated prevalence of 30-60% [1-3]. RBD is characterized by the enactment of dreams as a result of the loss of physiological atonia during REM sleep. Behaviors displayed include hitting, kicking, shouting but also laughing. The associated dreams are often violent or frightening in origin, and sometimes patients harm themselves or their bed partners with their movements [1]. Remarkably, the RBD-associated movements of PD patients are usually much faster, stronger and smoother than during the day, suggesting that the movements “bypass” the affected extrapyramidal systems [4].

To make a diagnosis of RBD, the current 2nd edition of the International Classification of Sleep Disorders (ICSD-II) requires the combination of clinical features (either by history or on nocturnal video recordings) and the presence of REM sleep without atonia as an electromyographic (EMG) finding during sleep recordings (Table 1) [5]. The gold standard for the diagnosis of RBD therefore entails a clinical interview, preferably by a sleep medicine specialist, together with at least one night of polysomnography with audiovisual recordings (video-PSG). However, referring every PD patient with complaints of nocturnal restlessness to a sleep medicine center is time-consuming, expensive and may not always be feasible in clinical practice. As a result, many movement disorder specialists base their diagnosis of RBD solely on the description of the typical behaviors by the bed partner of the patient. Studies show that this practice results in frequent misdiagnoses [6] and, consequently, overtreatment. Mimicking disorders such as obstructive sleep apnea, confusional arousals and nocturnal hallucinations should be excluded [7], especially because some of these may worsen with clonazepam, the first-line treatment of RBD. Therefore, there is a need for less expensive, easy to use methods to diagnose RBD.

**Table 1:** ICSD-II criteria for REM sleep behavior disorder

A	Presence of REM sleep without atonia; the EMG finding of excessive amounts of sustained or intermittend elevation of sub-mental EMG tone or excessive phasic submental or (upper or lower) limb EMG twitching.
B	At least one of the following is present:
	I. Sleep related injurious, or disruptive behaviors by history
	II. Abnormal REM sleep behaviors documented during PSG monitoring
C	Absence of EEG epileptiform activity during REM sleep unless RBD can be clearly distinguished from any concurrent REM sleep-related seizure disorder.
D	The sleep disturbance is not better explained by any other sleep disorder, medical or neurological disorders, mental disorders, medication use, or substance abuse

*EMG*, electromyography, *EEG*, electroencephalography, *PSG*, polysomnography, *RBD*, REM sleep behavior disorder, *REM*, rapid eye movement.

The REM sleep behavior disorder screening questionnaire (RBDSQ) was developed as an easy to use screening method [8,9]. The scale was created in German and English and more recently a Japanese version has been validated [10]. Although the questionnaire shows good internal consistency and a high sensitivity (96%) compared to the clinical interview, it has a low specificity (56%) [8]. The REM sleep behavior disorder questionnaire Hong Kong (RBDQ-HK) has been developed, tested and validated in Chinese patients based on the ICSD-II criteria [11]. It was validated in a group of PSG-confirmed RBD patients and controls. The overall RBDQ-HK score was significantly higher in the RBD group. ROC analysis showed that a cut off score of 18/19 had moderate sensitivity and specificity [11]. More recently Frauscher et al. published a validation study of the Innsbruck REM sleep behavior disorders inventory [12]. The scale had a sensitivity of 91.4% and a specificity of 85.7% for both idiopathic and PD related RBD (AUC, 0.886). Interestingly, the scores of patients sleeping alone were comparable with patients with a bed partner.

Actigraphy has been suggested as another possible diagnostic tool for RBD. Actigraphy may be a useful instrument to obtain general measures such as total sleep time, sleep efficiency and wake after sleep onset [13,14]. Compared to questionnaires, actigraphy should give a more objective representation of actual motor activity during the night. In addition, actigraphy is much less expensive and cumbersome compared to video-PSG and could be used in a home setting for several days, which may compensate for night-to-night fluctuations in the presence or severity of RBD symptoms. As such the use of actigraphy in the diagnosis of RBD seems attractive and the first results on its use are indeed promising. Naismith et al. found that PD patients with RBD had a higher number of bouts scored as “wake” by actigraphy, compared to patients without RBD, based on questionnaires [15]. In the current study, we sought to confirm these findings in a larger group of well-defined PD patients. We compared actigraphy outcomes in PD patients with and without RBD, based on the gold standard of a clinical interview in combination with video-PSG. Furthermore we searched for an optimal cut-off point to actually implement the use of actigraphy in clinical practice.

## Methods

### Design and study population

All patients were recruited from Sleep Medicine Centre Kempenhaeghe, a tertiary clinic for patients with sleep disorders in the Netherlands. At Kempenhaeghe, PD patients with sleep complaints are seen in a dedicated program, including an extensive clinical consultation followed by attended video-PSG that night. Referral reasons could be diverse e.g. insomnia, restless legs syndrome, sleep apnea, and thus did not only pertain to RBD. We included all consecutive idiopathic PD patients referred to the clinic as part of their regular care. All patients satisfied the UK Brain bank criteria for PD and were assessed using their usual medication. Data were collected as part of the regular medical care. The study was performed according to the guidelines of the Medical Ethical Committee of the Radboud University Nijmegen Medical Centre. All patients gave informed consent to use these data for further study.

#### Clinical characteristics

Demographic, clinical and disease characteristics were recorded. Disease stage was rated using the Hoehn & Yahr staging system [16]. The use of levodopa mono therapy, dopamine agonist mono therapy and combination therapies was registered. Overall dopaminergic treatment was quantified by calculating the Levodopa Equivalent Dose (LED) in mg/day [17]. In addition, nocturnal dopaminergic treatment was estimated by the dopaminergic dose taken before going to bed in LED (LED-night). The use of anti-depressants -which can cause or aggravate RBD- was actively asked for and listed.

#### RBD diagnosis

The diagnosis of RBD was made according to the ICSD-II criteria. During the clinical interview, the presence of movements and vocalizations during sleep was screened for by a sleep specialist experienced with PD (ML and SO). The interview was followed by one night of video-PSG. Sleep was scored by laboratory technicians highly experienced with scoring polysomnographic recordings in PD patients, and checked by a sleep medicine specialist (SO). The presence of REM sleep without atonia was determined by quantifying the EMG-signal of the m. submentalis using the “SinBar-group” criteria [18]. Tonic EMG activity was scored in 30 seconds epochs and was considered pathological if the amplitude was more than twice the background amplitude or exceeded 10 μV in more than 50% of the epoch. Phasic EMG activity was scored in 3 seconds mini-epochs and determined to be increased when it contained a burst of EMG activity lasting between 0.1 and 5.0 seconds with an amplitude exceeding twice the background activity. REM sleep without atonia was diagnosed when more than 18% of 3 second mini-epochs of REM sleep contained increased tonic and/or phasic EMG activity. Finally, the presence of abnormal REM sleep behaviors during PSG was determined using the synchronized video recordings.

#### Actigraphy

Actigraphy was performed using the Actiwatch system (Actiwatch AW4, Cambridge Neurotechnology Ltd, Cambridgeshire, United Kingdom), a piezoresistive uni-axial accelerometer. In agreement with previous studies, the recording device was placed on the wrist of the least affected site. Accelerometer signals were digitally sampled at a rate of 32 Hz. The actigraphy device is small, comfortable to wear and according to the patients did not interfere with their normal sleeping behavior. The first night of measurement was done simultaneously with the clinical PSG recording. Sleep and wake times were synchronized with PSG “lights off” and “lights on”. After the first night in the sleep center the actigraph was worn for seven consecutive nights at home. Actigraphy data were analyzed using Sleep Analysis 7.23 software (Cambridge Neurotechnology Ltd, Cambridgeshire, United Kingdom). Epoch length was set on 0.25 min. Outcome measurements were total sleep time, sleep efficiency, sleep latency, number and length of wake bouts and total and mean activity scores. Wake bouts during the sleep interval were defined as the total number of continuous blocks in an interval where the activity within the epoch was above the sleep threshold and therefore scored as “wake”. The wake threshold value (i.e. the number of activity counts used to define wake) was set to medium sensitivity, i.e. 40.0 activity counts per epoch.

#### Data analysis

Actigraphic measurements were compared between PD patients with and without the diagnosis of RBD. Comparisons were tested using independent t-test and chi-square test depending on the variable. As the Hoehn & Yahr stage was not normally distributed, a Mann- Whitney U test was used for this variable. Using Pearson’s correlation coefficients, associations were analyzed between the number of bouts classified as wake by actigraphy and actual wake time during the PSG recording. Multiple regression analysis was used to correct for actual wake time according to the PSG in the estimation of the number of bouts classified as wake by actigraphy. The diagnostic accuracy of the number of wake bouts as a diagnostic tool for the presence of RBD, was assessed using ROC analyses. Sensitivity, specificity and positive predictive value were calculated. Missing values were <5%, therefore all percentages are presented as valid percentages. All data are shown as mean ± SD or N (%). All results are based on two-tailed tests, with a significance level set at p < .05.

## Results

### Study population

During the study period, 54 PD patients were included. Nine patients were excluded from the analysis, one because of technical problems and eight because they had less than 10 minutes of REM sleep during the PSG. Twenty-three of the 45 remaining patients (51.1%) were diagnosed with RBD according to the ICSD-II criteria.

### Clinical and disease characteristics

In Table 2, clinical demographic as well as disease characteristics of the study subjects are summarized. Patients with RBD (PD + RBD) were older than those without (PD-RBD). Disease severity was higher in patients with RBD with longer disease duration and more advanced H&Y disease stage (Table 2). PD + RBD patients used higher doses of dopaminergic treatment. In the PD + RBD group ten patients used levodopa only and thirteen patients used a combination of levodopa and a dopamine agonist. Three patients in the PD-RBD group did not use medication, three were on levodopa monotherapy, four on dopamine agonist monotherapy, and twelve used a combination of levodopa and a dopamine agonist. Use of anti-depressants was not different between groups.

**Table 2:** Clinical and disease characteristics

	***RBD+***	***RBD-***	***p***
N	23	22	
Men (%)	17 (73.9)	13 (59.1)	.292
Age (yr)	64.3 ± 9.4	58.1 ± 8.8	. ***028***
**Disease characteristics**			
Disease duration (yr)	9.5 ± 6.4	4.3 ± 2.8	***.024***
LED (mg/day)	1089.4 ± 582.9	697.7 ± 563.1	***.027***
LED night (mg/day)	117.2 ± 51.8	140.6 ± 40.9	.298
Hoehn & Yahr (%)			***.025***
1	-	2 (9.1)	
1.5	-	4 (18.2)	
2	16 (69.6)	13 (59.1)	
2.5	4 (17.4)	-	
3	3 (13.0)	3 (13.6)	
**Use of anti-depressants**			
SSRI (%)	4 (17.4)	4 (18.2)	.945
TCA (%)	-	2 (9.1)	.139
Other (%)	3 (13.0)	-	.080

Results are mean ± SD, except if otherwise specified.

*LED*, Levodopa equivalent dose.

LED night Levodopa Equivalent Dose taken before going to bed.

*SSRI*, selective serotonin reuptake inhibitors.

*TCA,* tricyclic antidepressants.

Bold p values are significant.

### Sleep parameters

Objective sleep quality in PD + RBD patients was lower; patients with RBD spent more time awake, and had a shorter total sleep time and lower sleep efficiency (Table 3). The prevalence of sleep disorders other than RBD was not different between groups (Table 3).

**Table 3:** Sleep parameters

	**RBD+**	**RBD-**	**p**
N	23	22	
**PSG results**			
Total sleep time (min)	330.0 ± 51.6	375.6 ± 65.9	***.008***
Sleep efficiency (%)	67.9 ± 8.7	76.3 ± 10.7	***.004***
Sleep latency (min)	13.8 ± 12.2	10.1 ± 8.1	.138
% N1	15.2 ± 8.0	10.3 ± 5.2	***.037***
% N2	58.9 ± 11.3	58.0 ± 10.9	.677
% N3	13.0 ± 11.3	15.7 ± 9.5	.304
% REM	13.0 ± 8.3	15.9 ± 7.2	.118
% Wake	30.0 ± 9.4	22.6 ± 10.9	***.003***
Awakenings (no.)	35.3 ± 14.5	28.9 ± 11.1	.109
PLM index	36.2 ± 47.2	21.7 ± 31.9	.138
**Other sleep diagnoses**			
Insomnia (%)	18 (78.3)	18 (81.8)	.766
OSAS (%)	6 (26.1)	9 (40.9)	.292
RLS (%)	6 (26.1)	6 (27.3)	.928
Hypersomnia (%)	12 (52.2)	8 (36.4)	.286

Results are mean ± SD, except if otherwise specified.

% N1(N2, N3, REM) = percentage in stage N1 (N2, N3, REM) sleep), *PLM*, periodic limb movement; *OSAS*, obstructive sleep apnea syndrome; *RLS*, restless legs syndrome.

Bold p values are significant.

### Actigraphy

Actigraphy results are presented in Table 4, both during the first night (i.e. simultaneous with the video-PSG) and for the eight consecutive nights in total. In contrast to the PSG results, total sleep time and sleep efficiency as estimated by actigraphy were not different between patients with and without PD, both during the first night and overall eight nights. The total number of bouts classified as wake was 30% higher in the PD + RBD compared to PD-RBD group (PD + RBD 73.2 ± 40.2 vs. PD-RBD 48.4 ± 23.3, p = .016). This was not the case for the length of the wake bouts. Total activity and mean activity scores during sleep were not different either. As several clinical characteristics differed between patients with and without PD, comparisons were repeated using multiple regression analyses corrected for these variables, however this did not change the results. Although a correlation was found between number of bouts classified as wake and actual wake time (r = .31, p = .039), regression analysis correcting for actual wake time still showed a significant effect of the presence of RBD on the number of wake bouts (R-squared = 0.18, standardized-beta = 0.31, p = .043).

**Table 4:** Actigraphy results

	**RBD+**	**RBD-**	**p**
N	23	22	
**Actigraphy night 1**			
Total sleep time (min)	396.5 ± 72.0	429.1 ± 58.4	.103
Sleep efficiency	82.7 ± 12.3	87.6 ± 8.7	.133
Sleep latency (min)	3.3 ± 6.5	2.4 ± 4.7	.580
No wake bouts	78.4 ± 46.1	49.5 ± 22.2	***.011***
Length wake bouts (min)	1.1 ± 0.5	1.2 ± 0.6	.488
Total activity score	12943.8 ± 11432.2	10132.6 ± 6847.6	.325
Mean activity score	6.9 ± 5.7	5.4 ± 3.9	.299
**Actigraphy mean of 8 nights**			
Total sleep time (min)	397.4 ± 91.1	389.5 ± 64.1	.738
Sleep efficiency	78.4 ± 14.6	84.7 ± 9.5	.097
Sleep latency (min)	10.0 ± 10.6	5.8 ± 10.3	.187
No wake bouts	73.2 ± 40.2	48.4 ± 23.3	***.016***
Length wake bouts (min)	1.3 ± 0.6	1.4 ± 0.5	.689
Total activity score	17885.2 ± 14375.8	12613.9 ± 9793.2	.160
Mean activity score	10.4 ± 11.5	7.2 ± 5.4	.231

Results are mean ± SD, except if otherwise specified.

% N1(N2, N3, REM) = percentage in stage N1 (N2, N3, REM) sleep), *PLM*, periodic limb movement; *OSAS*, obstructive sleep apnea syndrome, *RLS*, restless legs syndrome.

Bold p values are significant.

### Actigraphy in clinical practice

**Figure 1:**
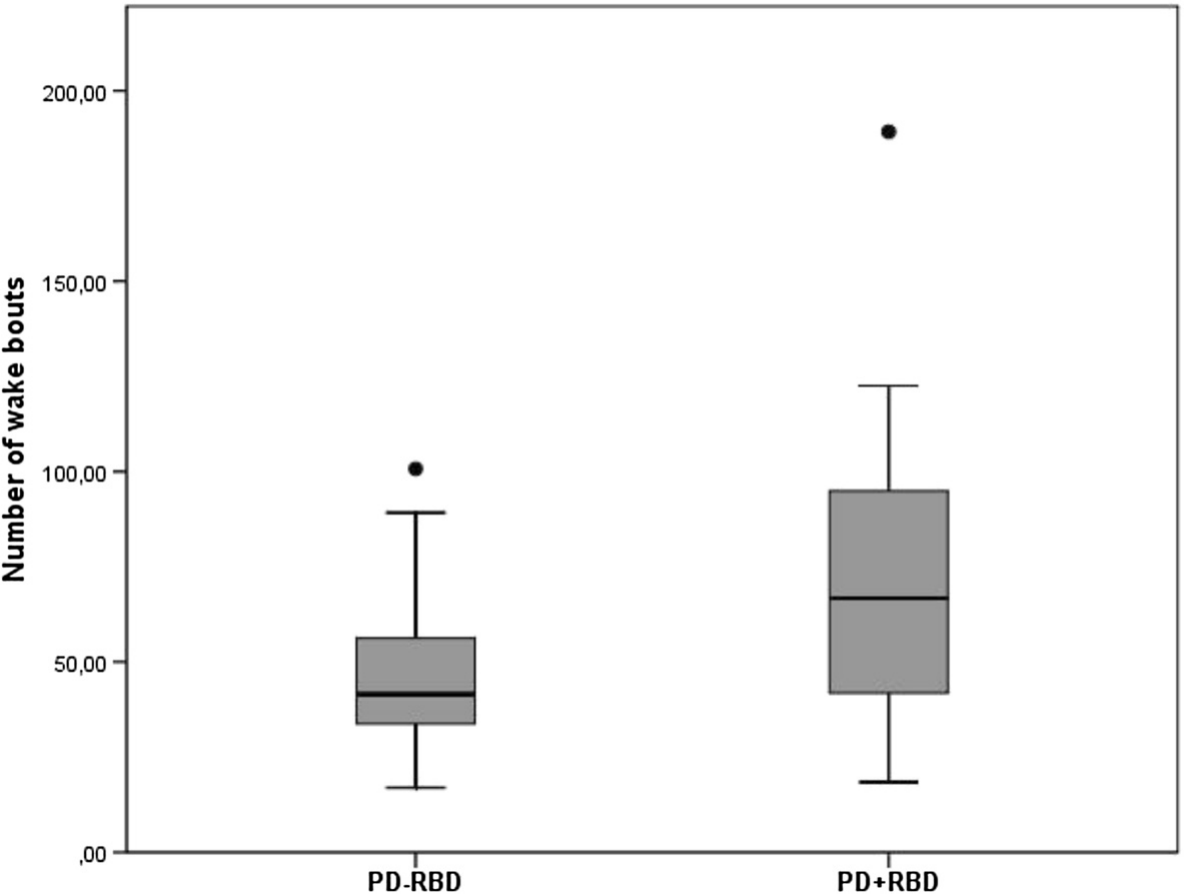
**Distribution of wake bouts across groups.** Boxplot of number of wake bouts per night measured over 8 nights in PD with without and with RBD.

**Figure 2:**
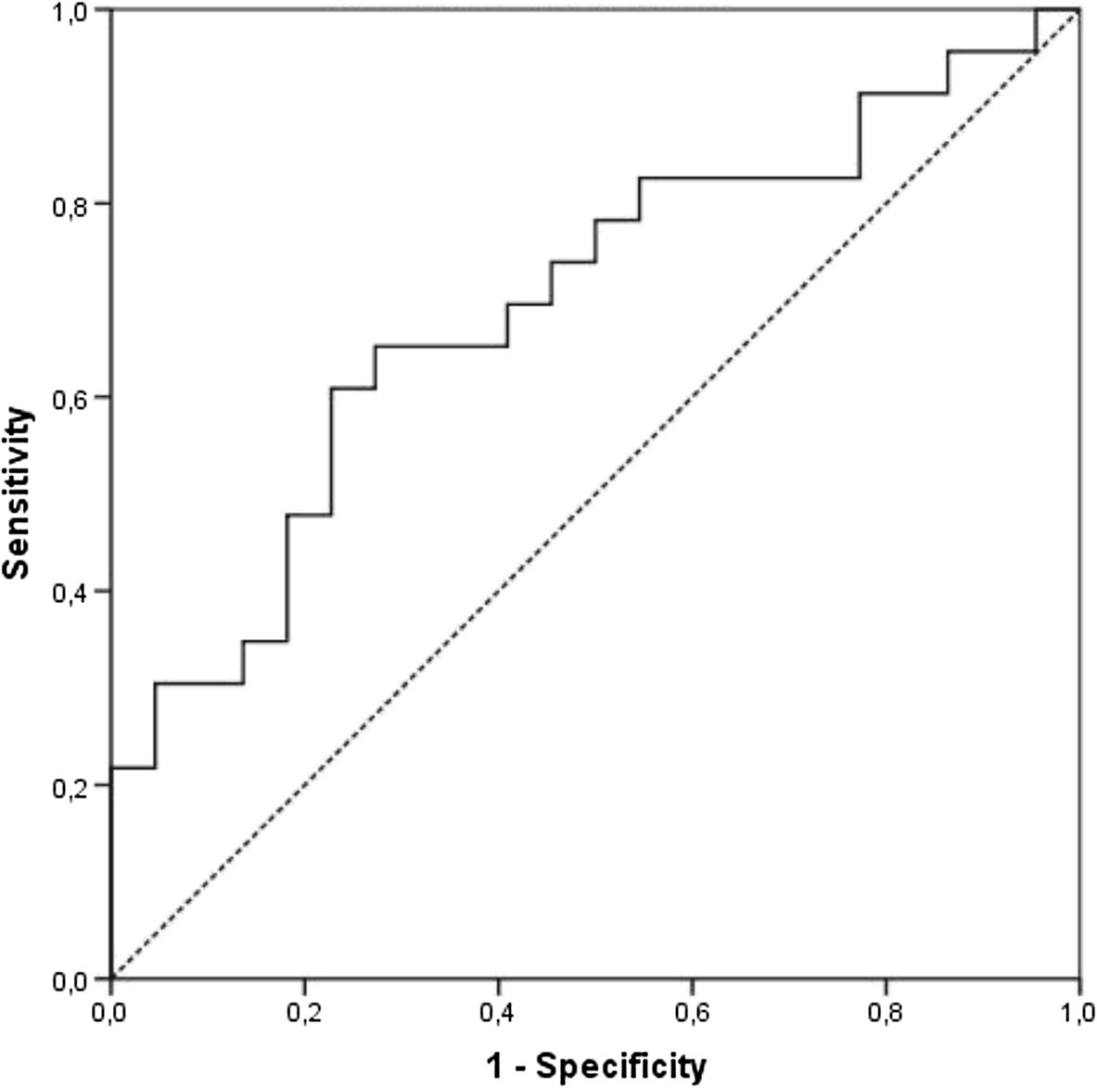
**ROC curve using number of wake bouts in diagnosis of RBD.** Area under the curve (AUC) = 0.696.

**Figure 3:**
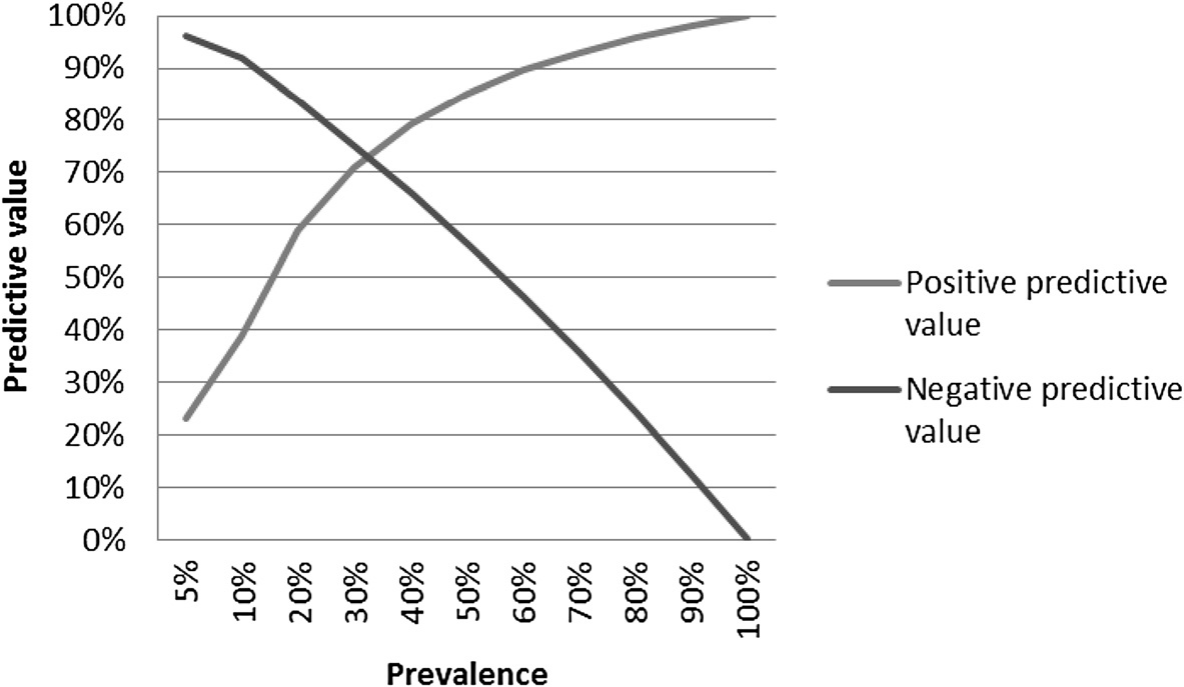
Predictive value of actigraphy for RBD in PD, in relation to RBD prevalence.

To study the clinical relevance of the previous findings, we calculated the sensitivity, specificity and the positive and negative predictive value of actigraphy for the diagnosis of RBD. Figure 1 shows the distribution of wake bouts in the PD + RBD and PD-RBD groups. A cut-off of 95 wake bouts per night yielded a specificity of 95.5%, a sensitivity of 26.1%, a positive predictive value of 85.7% and a negative predictive value of 55.3%. Figure 2 shows the ROC curve when using different cut-offs for the number of wake bouts during the eight measurement nights. The area under the curve was 0.696 with a significance of p = .025. Figure 3 shows the resulting positive and negative predictive values according to different prevalence rates of RBD; which in our cohort was 51.1%.

We additionally looked at the final diagnosis for patients where the clinical interview was incongruent with the final diagnosis of RBD. Based on the clinical interview, seven patients were suspected of having RBD, but did not fulfill the full ICSD-II criteria. All but one of these patients had sleep initiation or maintenance problems (insomnia); two were diagnosed with obstructive sleep apnea syndrome, two had restless legs syndrome, three showed an increased level of periodic leg movements, and one suffered from nocturnal hallucinations (Table 5). Six of the patients had a wake bout count lower 95 (Table 5). Seven patients did not have a clinical history of RBD, but PSG findings allowed an RBD diagnosis according to the ICSD-II criteria. In these patients there was either no bed partner, or the bed partner claimed to be always fast asleep not noticing any abnormal behavior of the patient. Additional sleep diagnoses in this group are listed in Table 5. The actigraphy-based number of wake bouts in this group was highly diverse, showing no consistent direction.

**Table 5:** Mismatch between RBD diagnosis based on clinical interview and ICSD II criteria

**PD patients with clinical interview suspicious for RBD but not confirmed by ICSD II criteria**		
***Study number***	*Final diagnoses*	***Number of wake bouts according to actigraphy***
**21**	OSAS	52.4
**24**	Insomnia, PLMD	100.7
**29**	Insomnia, PLMD, hallucinations	19.4
**32**	Insomnia, RLS	56.3
**48**	Insomnia	28.0
**71**	Insomnia, RLS, PLMD	83.5
**73**	Insomnia, OSAS	42.1
**PD patients with clinical interview negative for RBD but with diagnosis based on ICSD II criteria**		
***Study number***	*Other diagnoses next to RBD*	*Number of wake bouts according to actigraphy*
**1**	Insomnia, OSAS, RLS	108.1
**12**	Insomnia, RLS, PLMD	92.3
**16**	Insomnia	59.8
**44**	Insomnia	25.7
**45**	Insomnia, RLS, PLMD	189.3
**65**	Insomnia, RLS	32.9
**75**	Insomnia, PLMD	71.5

*OSAS*, obstructive sleep apnea syndrome, *PLMD*, periodic leg movement disorder, *RLS*, restless legs syndrome.

## Discussion

Solely using the clinical interview to assess the possible presence of RBD in PD patients, often results in misdiagnoses. However, even judicious use of video-PSG is costly and not always feasible. Therefore, there is a clear need for new screening tools for RBD. Our results show that using actigraphy, the number of bouts classified as “wake” is significantly higher in PD patient with RBD compared to PD patients without. Accordingly, we show that actigraphy has a very high specificity and a good positive predictive value for diagnosing RBD in PD patients.

Wake bouts as scored by actigraphy were previously suggested as a possible useful marker in the diagnostic workup of RBD in PD: our findings are in agreement with Naismith et al., who studied 22 patients with 14 consecutive nights of actigraphy [15]. However, the actual number of wake bouts was almost twice as high in our patient group, compared to theirs. Since sensitivity settings of the actigraphs were the same, this difference may have been caused by different epoch length settings, which was 0.25 min in our study and 0.50 in the study of Naismith et al. [15]. In addition, we used video-PSG in combination with a clinical interview by a sleep medicine specialist as the gold standard for the diagnosis of RBD, instead of questionnaires.

Previous studies have suggested that actigraphy is an useful method to measure sleep quality in PD patients. Correlations were found between actigraphy and total sleep time, wake after sleep onset and subjective complaints about nocturnal sleep [13,14]. Our results however showed a difference between total sleep time and sleep efficiency measured with actigraphy and PSG. Although the actigraph was measured on the least affect side, we cannot exclude that the presence of tremor, on-off fluctuations and/or dyskinesias may have influenced the results. More research is needed to study the influence of PD motors symptoms on actigraphic results during the night.

There was no increase in either total or mean activity levels during sleep, which could have been expected in patients with REM related movements. However, as RBD associated movements lead to activity well above the threshold that is represented as “sleep” by actigraphy, they are almost always scored as “wake bouts” rather than increased activity during sleep. Variables other than the presence of RBD may have influenced the number of bouts classified as wake. The periodic limb movement index was, although not significantly, higher in the PD + RBD group compared to the PD-RBD group. The lack of significance could be caused by a large difference in variance. Periodic limb movements can cause sleep disturbances and therefore increase the number of wake bouts. Our groups were not matched with respect to age, disease duration, disease stage and medication use, and these factors may also influence sleep. However, regression analyses correcting for these clinical characteristics and PSG-determined actual wake time during the night, still showed a significant differences in the number of wake bouts between groups. These findings suggest that the increased number of wake bouts is primarily the result of the presence of RBD.

Results showed that using an epoch length of 0.25 min and a cut-off of 95 wake bouts per night, actigraphy is a highly specific tool for RBD in PD patients, albeit with a low sensitivity. As the prevalence of RBD in PD ranges between 30% and 60% [1-3], actigraphy has a positive predictive value between 70 and 90% which is reasonable. Based on a semi-structured clinical interview alone, we found seven patients incorrectly suspected of having RBD. Of these, only one patient scored above the threshold of 95 wake bouts per night. Therefore, these results show an additional value of using actigraphy next to a clinical interview in the diagnostic trajectory of RBD. Seven patients had no clinical history of RBD-like behavior but still fulfilled the diagnostic PSG criteria of RBD, and actigraphy did not differentiate these patients from the group without RBD. Actigraphy therefore mainly has a role in combination with at least a clinical suspicion of RBD, rather than a screening instrument in PD patients without complaints of RBD. Actigraphy should not be used in the diagnosis of idiopathic RBD: although clear studies about the prevalence of RBD in the general elderly population are lacking, rates are estimated between 0.38% and 0.50%, leading to a positive predictive value below 5% [19,20].

Contrary to the high specificity and low sensitivity of actigraphy, previous research showed that RBD questionnaires have a high sensitivity and a somewhat low specificity [8,10-12]. Combining actigraphy and RBD questionnaires could therefore lead to a more accurate diagnosis of RBD. The combination of these two tools could reduce the need for video-PSG even more. Future research should focus on the clinical value of using a combination of both methods.

Our study used the ICSD-II criteria for the diagnosis of RBD. These criteria are not unambiguous unfortunately. They include presence of atonia during REM sleep, which represents a pathological increase of either phasic EMG activity, tonic EMG activity or both. Cut-off points to diagnose pathological increased phasic and tonic EMG activity are not mentioned in the criteria however, and no agreement has been reached on this point among international research groups. Here, we therefore adopted the criteria developed by the SinBar group, although several other visual and computerized scoring methods have been mentioned in literature [2,18,21-26].

## Conclusions

PD patients with RBD showed a significantly higher number of bouts scored as “wake” using actigraphy, compared to patients without RBD. In clinical practice, actigraphy has a high specificity, but low sensitivity in the diagnosis of RBD. According to our results and previous studies on the use of RBD questionnaires, the combination of both tools could be a promising method to diagnose RBD in PD patients, leading to a decrease in the need for the costly and time-consuming video-PSG.

## Competing interests

BRB was supported by a VIDI research grant from the Netherlands Organization for Scientific Research (grant no. 016.076.352). SO was supported by a VIDI research grant from the Netherlands Organization for Scientific Research (grant no. 016.116.371). The other authors have no conflict of interest.

## Authors’ contributions

ML: conception and design, acquisition of data, analysis and interpretation of data, drafting of the manuscript, final approval for publishing. JA: conception and design, interpretation of data, critical review of manuscript, final approval for publishing. BB: conception and design, critical review of manuscript, final approval for publishing. SO: conception and design, acquisition of data, interpretation of data, drafting of the manuscript, final approval for publishing. All authors read and approved the final manuscript.

## Pre-publication history

The pre-publication history for this paper can be accessed here:

http://www.biomedcentral.com/1471-2377/14/76/prepub
